# Temporary disruption of the blood-brain barrier using an implantable ultrasound system for recurrent glioblastoma patients under IV carboplatin chemotherapy: initial phase 1/2a clinical trial observations

**DOI:** 10.1186/2050-5736-3-S1-O14

**Published:** 2015-06-30

**Authors:** Alexandre Carpentier, Michael Canney, Alexandre Vignot, Catherine Horodyckid, Lauriane Goldwirt, Delphine Leclercq, Jean-Yves Delattre, Jean-Yves Chapelon, Ahmed Idbaih

**Affiliations:** 1 Pitié-Salpêtrierè Hospital, Assistance Publique – Hôpitaux de Paris, Paris, France; 2 CarThera, Lyon, France; 3 Hôpital Saint-Louis, Assistance Publique - Hôpitaux de Paris, Paris, France; 4 Inserm, Lyon, France

## Background/introduction

One of the main limitations to the efficacy of chemotherapy in the brain is the blood-brain barrier (BBB). Previous pre-clinical studies have demonstrated that disruption of the BBB using pulsed ultrasound in combination with an ultrasound microbubble contrast agent can significantly increase the concentration of chemotherapy agents in the brain. Our group has developed an implantable, MR compatible ultrasound device for temporarily disrupting the BBB. The safety of repeated disruption of the BBB using such a device was previously demonstrated in a long-term safety study in four non-human primates. The purpose of this work was to determine the safety and potential efficacy of temporarily disrupting the BBB in patients with recurrent glioblastoma before chemotherapy administration in a first-in-man clinical trial.

## Methods

A Phase 1/2a clinical trial to open the BBB in recurrent glioblastoma patients was approved to start in July 2014 at the Hospital Pitie Salpetriere in Paris, France.

Participating patients will be implanted with an 11.5-mm diameter biocompatible 1 MHz ultrasound transducer, which will be fixed to the skull bone in a standard burr hole, after which the skin will be closed. Once a month, the device will be connected to an external generator system using a transdermal needle connection, and patients will receive a two minute pulsed ultrasound sonication (25,000 cycles/burst, 1 Hz) in combination with systemic administration of an ultrasound contrast agent. BBB disruption will be monitored immediately after sonication using dynamic T1-weighted MR imaging using a gadolinium based MR contrast agent to measure the permeability coefficient, Ktrans, to quantify the magnitude of BBB disruption. Systemic intravenous injection of Carboplatin (Area Under the Curve 4-6) will be delivered immediately following MR imaging. Patients will follow a progression of ultrasound dose in which the pressure is increased from 0.5 to 0.8 MPa throughout the course of the study.

## Results and conclusions

Pre-clinical results in a non-human primate demonstrated an increase in concentration of carboplatin of 200-600% after ultrasound-induced disruption of the BBB. Note that this clinical trial was approved to begin in July 2014 and thus no results were yet available in patients at the time of abstract submission. The most recent results will be presented at the meeting. These studies will verify that the BBB can be safely disrupted in patients with recurrent glioblastoma and will evaluate the tolerance and potential efficacy of such an approach in combination with intravenous administration of a chemotherapy regimen of carboplatin.

